# The Origins and Generation of Cancer-Associated Mesenchymal Stromal Cells: An Innovative Therapeutic Target for Solid Tumors

**DOI:** 10.3389/fonc.2021.723707

**Published:** 2021-08-26

**Authors:** Wei Li, Jin Yang, Ping Zheng, Haining Li, Shaolin Zhao

**Affiliations:** ^1^Center of Research Laboratory, Department of Laboratory Medicine, The First People’s Hospital of Lianyungang, Lianyungang, China; ^2^Department of Clinical Laboratory Diagnostics, Kangda College of Nanjing Medical University, Lianyungang, China

**Keywords:** mesenchymal stromal cells, solid tumor, tumor microenvironment, malignant transition, generating process

## Abstract

Cancer-associated mesenchymal stromal cells (CA-MSCs) have been isolated from various types of tumors and are characterized by their vigorous pro-tumorigenic functions. However, very little is known about the origins and generating process of CA-MSCs, which may facilitate the identification of biomarkers for diagnosis or innovative targets for anti-cancer therapy to restrain the tumor growth, spread and chemotherapy resistance. Current evidences have indicated that both distally recruited and local resident MSCs are the primary origins of CA-MSCs. In a tissue type-dependent mode, tumor cells together with the TME components prompt the malignant transition of tumor “naïve” MSCs into CA-MSCs in a direct cell-to-cell contact, paracrine or exosome-mediated manner. In this review, we discuss the transition of phenotypes and functions of naïve MSCs into CA-MSCs influenced by tumor cells or non-tumor cells in the TME. The key areas remaining poorly understood are also highlighted and concluded herein.

## Introduction

Mesenchymal stromal cells, also known as mesenchymal stem cells (MSCs), are multipotent progenitor cells existing in different tissues (1), such as bone marrow, adipose, umbilical cords and brain. In injured tissues, MSCs have the ability to migrate specifically into the damaged tissues and participate in tissue regeneration (2, 3). These stromal cells are known to differentiate into osteocytes, adipocytes, chondrocytes and fibroblasts. Since tumors are considered as “wounds that do not heal”, MSCs can also be recruited specifically into tumor tissues in response to “cues” from tumor cells and the tumor microenvironment (TME) (4).

In the TME of neoplasia, MSCs are one of the key stromal cells, which are reported to play complex and dynamic roles in the pathophysiology of solid tumors (5, 6). Interactions with tumor cells and other components in the TME cause “naïve” MSCs to undergo genetic and functional changes, giving rise to the generation of tumor-promoting cancer-associated MSCs (CA-MSCs) (7, 8). Under the tumor-mediated “education”, CA-MSCs have displayed outstanding roles in the progress of tumor initiation, promotion, progression or metastasis (9, 10).

Generally, CA-MSCs still retain the differentiation capacity and stromal surface markers of the counterpart MSCs, and use the similar mechanisms to support tumor progression (7, 11). Within the confines of the TME, CA-MSCs have been demonstrated to play key roles in the generation of tumor supporting niche (12), in the promotion of neovascularization (13), and in the stimulation of epithelial-mesenchymal transition (EMT) in tumor cells (14, 15). They also contribute to tumor pathophysiology *via* increasing the proliferation and survival of tumor cells (16), or enriching the population of cancer stem-like cells (CSCs) in the TME (17). However, CA-MSCs possess several unique properties and a more potent pro-tumorigenic function *versus* their parental MSCs (18, 19). For instance, CA-MSCs display a stronger ability to differentiate into carcinoma-associated fibroblasts (CAFs) than naïve MSCs (20). There is also evidence that CA-MSCs display higher immunosuppressive and angiogenic properties than the parental MSCs (19, 21). However, the origins and generating process of CA-MSCs are still less studied and have not yet been well characterized.

Hitherto, multiple reports have demonstrated that CA-MSCs can be picked up from malignant tissues of patients with several types of solid tumors, including gastric cancer (22), ovarian cancer (9), lung cancer (23), colorectal cancer (24), lymphoma (25) and prostate cancer (26). Generally, CA-MSCs could be separated out from tumor tissues by the following methods, including: (i) direct tissue piece adhesion (15, 22), (ii) tissue piece digestion by protease solution (23, 24), and (iii) immunomagnetic separation of the digested cells (25). The identifications of CA-MSCs depend on the morphology, immunophenotype and multilineage differentiation capacities of the isolated cells, which is similar to the counterpart MSCs. In the TME, CA-MSCs clearly influence the formation and context of TME and consequently play a pro-tumorigenic role in tumor growth and spread (27). Herein, we highlight the potential origins of CA-MSCs, and review the malignant transformation of naïve MSCs into CA-MSCs influenced by tumor cells or non-tumor cells in distinct modes.

## Different Properties of CA-MSCS *Versus* Naïve MSCS

MSCs are a heterogeneous population of progenitor cells, which play an active role in tumor progression (28). Upon sensing the “cues” from tumor, MSCs continuously migrate into local tumor sites and incorporate into the integral components of tumor stroma (7, 28). After affected by tumor cells and the surrounding microenvironment, the newly arrived naïve MSCs can be altered in their properties and converted into a pro-tumorigenic population termed as “CA-MSCs” (29). In the TME, CA-MSCs interact with the non-MSCs stromal populations and help to establish a favorable niche for tumorigenesis, metastasis, angiogenesis or drug resistance.

As increasingly demonstrated in the literature, CA-MSCs play a key role in tumor promotion via: (i) influencing the innate and adaptive immune systems to suppress immune response; (ii) promoting the survival and growth of tumor cells by producing growth factors, chemokines and cytokines; (iii) promoting tumor angiogenesis by secreting angiogenic factors or differentiating into endothelial cells; and (iv) promoting tumor cell metastasis by producing chemokines or enhancing EMT in tumor cells (30). However, accumulating evidence has indicated the multiple differences of CA-MSCs from the counterpart naïve MSCs in several aspects (18, 27), which are displayed in details as following:

### CA-MSCs Play a More Potent Pro-Tumorigenic Role Than Naïve MSCs

A study in gastric cancer revealed that gastric cancer-derived MSCs (GC-MSCs) promote gastric cancer growth and progression more efficiently than bone marrow-derived MSCs (BM-MSCs) do *via* a considerable secretion of IL-8 (19). Tumor-resident GC-MSCs were proved to facilitate the proliferation and migration of gastric cancer cells more potently than BM-MSCs, and to exhibit a higher ability of pro-angiogenesis compared to BM-MSCs. Another study in lymphomas also showed that MSCs isolated from spontaneous lymphomas (L-MSCs) strikingly enhance tumor growth in comparison to BM-MSCs (25). Furthermore, L-MSCs were observed to result in greater recruitment of monocytes, macrophages, and neutrophils to tumor tissue than BM-MSCs. Likewise, breast cancer-derived MSCs were also isolated and demonstrated to have a greater potential to promote breast cancer cell growth and decrease apoptosis upon exposure to cisplatin compared to BM-MSCs (31).

### The Expression Profile of CA-MSCs Is Different From Naïve MSCs

The expression profile of cytokines or chemokines in CA-MSCs has been changed after the malignant conversion (**Table 1**), which may be responsible for their predominant roles in tumor promotion. It was reported that CA-MSCs isolated from human ovarian carcinoma increase the number of CSCs and promote tumor growth more efficiently than the control MSC *via* an increased production of BMP family (32). Another study in breast cancer discovered that IL-6 is significantly higher secreted by CA-MSCs than BM-MSCs, which mediates the pivotal role of CA-MSCs in enhancing the proliferation of tumor cells (31). In addition, CCR2 ligands including CCL-2, CCL-7 and CCL-12 were shown to be markedly higher expressed in tumor-infiltrating L-MSCs than in BM-MSCs (25). Although L-MSCs behave not differently from BM-MSCs in their effects on adaptive immune cells, they are more effective in recruiting monocytes/macrophages *via* CCR2, which positions L-MSCs a much stronger tumor-promoting population than BM-MSCs. Besides, a study in lung carcinoma also clarified that CA-MSCs display a transcriptome distinct from that of the non-tumoral adjacent tissue-derived MSCs (N-MSCs) (33). They selectively promote dissemination of the primary lung cancer cells rather than local growth, and possess a phenotype distinct from that of N-MSCs, which can not display a comparable metastasis-promoting ability. Additionally, miRNA profile has also been reported to be differentially expressed in GC-MSCs relative to adjacent non-cancerous tissue-derived MSCs (34). In GC-MSCs and cancer tissues, miR-214, miR-221 and miR-222 were found to be commonly upregulated, which are tightly associated with lymph node metastasis, venous invasion and the TNM stage (34) (**Table 1**).

**Table 1:** Increased expression profiles in CA-MSCs compared to naïve MSCs.

Tumor Types	Expression Profile	Regulatory Signal	Reference
Ovarian carcinoma	BMP2, BMP4, BMP6	BMP	(32)
Breast cancer	IL-6	IL-6/STAT3	(31)
Lymphoma	CCL-2, CCL-7, CCL-12, IL-5, IL-6, CXCL-10, V-EGF	CCL2/CCR2	(25)
Lung carcinoma	TGF-β, IL-6, GREM1, LOXL2, SRGN, THBS2, IGF2	TGF-β	(33)
Gastric cancer	miR-214, miR-221, miR-222	Akt/mTOR	(34)

### CA-MSCs Exhibit Greater Proliferative and Migratory Capacities Than Naïve MSCs

It was reported that CA-MSCs have shorter doubling times than naïve MSCs, which might be due to the higher expressions of proliferation-related genes, such as murine double minute 2 and p21, the zinc finger transcriptional factor sal-like protein 4, in CA-MSCs compared to naïve MSCs (35). In addition, GC-MSCs were shown to possess particularly stronger migratory capabilities than their counterpart naïve MSCs (35).

### CA-MSCs Possess Stronger Immunosuppressive Activity Than Naïve MSCs

Compared to naïve MSCs, GC-MSCs could promptly promote the polarization of macrophages into an M2-like subtype, which displays an immunosuppressive activity than M1 subtype (15). Moreover, Luminex assay analysis demonstrated that the concentrations of pro-inflammatory cytokines TNF-α, IP-10, RANTES, and MIP-1α significantly decreased in the supernatant of macrophages after co-cultured with GC-MSCs (15). Another study clarified that MSCs derived from breast cancer tissues express higher levels of the immunosuppressive factors IL-4, IL-10 and TGF-β1 than MSCs isolated from normal breast tissues do (36). In addition, co-culturing peripheral blood mononuclear cells with breast cancer-derived MSCs increased the proportion of CD4^+^CD^25hi^Foxp^3+^ regulatory T cells (36).

### Other Differences Between CA-MSCs and Naïve MSCs

CA-MSCs have been shown to have a much greater ability to differentiate into tumor-supporting CAFs than naïve MSCs in the TME (20). In addition, a study in neuroblastoma demonstrated an increased number of the G0-G1 phase cells in CA-MSCs *versus* the counterpart MSCs, suggesting an essential role of CA-MSCs in regulating cancer dormancy (11). Compared to naïve MSCs, CA-MSCs also exhibit different adipogenic differentiating and immunoregulatory capabilities (33). Therefore, CA-MSCs have recently been considered as an attractive target for anti-cancer therapeutics or prognosis in various solid tumors.

## Multiple Origins of CA-MSCS

In inflammatory diseases, MSCs have been unveiled to display tropism for the inflammatory sites and release a broad repertoire of soluble factors to modulate immune response (37). Likewise, similar tropism of MSCs is observed within various solid tumors in response to the “cues” from tumor tissues (38–40). After engaging into the TME, the recruited MSCs can “evolve” into CA-MSCs and participate in the formation of tumoral niche facilitating for tumorigenesis, spread or immune escape. In view of this, as the initial step of MSC-mediated tumor progression, the tropism or migration of MSCs toward tumor sites is a pivotal process and the two main origins have hitherto been discovered for CA-MSC storage in the TME.

### Circulating BM-MSCs

The circulating BM-MSCs have been well established to be an important source of tumor resident CA-MSCs (41). Evidences for the tumor tropism or homing of BM-MSCs are obtained from both *in-vitro* and *in-vivo* investigations. On one hand, mounting studies demonstrated that co-cultured with tumor cells significantly promotes the migration of BM-MSCs, indicating a strong chemotactic function of tumor cells *via* secreting molecular factors (42, 43), such as transforming growth factor-β (TGF-β) and platelet derived growth factor (PDGF). On the other hand, the direct evidences for BM-MSCs’ migration seem to be clarified by *in-vivo* studies, in which bone marrow from GFP-transgenic mice were injected in a mouse model with tumor and a large proportion of GFP^+^ CA-MSCs can be found within tumor tissues (44).

Chemokines and cytokines secreted by both tumor cells and the surrounding stroma have been shown to be involved in the mechanisms underlying the migration or homing of the circulating BM-MSCs into the TME. It is well known that CCL2, CCL5, CXCL12 and CXCL16 produced by the tumor are crucially involved in the tropic process of BM-MSCs (38). These chemokines act as ligands for receptors expressed by the circulating BM-MSCs. In addition, cytokines including PDGF, TGF-β, vascular endothelial growth factor (VEGF) and tumor necrosis factor-α (TNF-α) have also been found to play an effective role in BM-MSC homing (45–47). For instance, a study in prostate carcinoma demonstrated TGF-β1 as a key molecule for regulating distantly recruitment of BM-MSCs into tumor sites (48).

### MSCs Resident in the Adjacent Normal Tissue of Tumor

In addition to circulating BM-MSCs, the adjacent normal tissue-resident MSCs have been demonstrated to be the most likely source of CA-MSCs in tumor tissues (33, 49). BM-MSCs display the phenotypes and functions, which are more distinct from those of CA-MSCs than the adjacent normal tissue-resident MSCs do (33). Moreover, the population of MSCs is notably more abundant in tumor sites than in the adjacent normal tissues in a various types of solid tumors (50). Thus, the adjacent tissue-resident MSCs are proposed as the primary origin or source of CA-MSCs. Upon receiving “cues” from tumor cells and non-tumor cells in the TME, naïve MSCs resident in the adjacent normal tissues may migrate and transplant into the tumor sites, which subsequently incorporate to the cohort of CA-MSCs (51). MSCs resident in adjacent normal tissues express homing profiles in response to inflammation signals or “cues” from tumor cells, thereby anchoring into the tumor bulks (51). However, the signaling pathways and the underlying mechanisms for MSC migration from the adjacent normal tissues into tumor site have not yet been clarified, although it has been regarded as the primarily origin of CA-MSCs.

**Figure 1:**
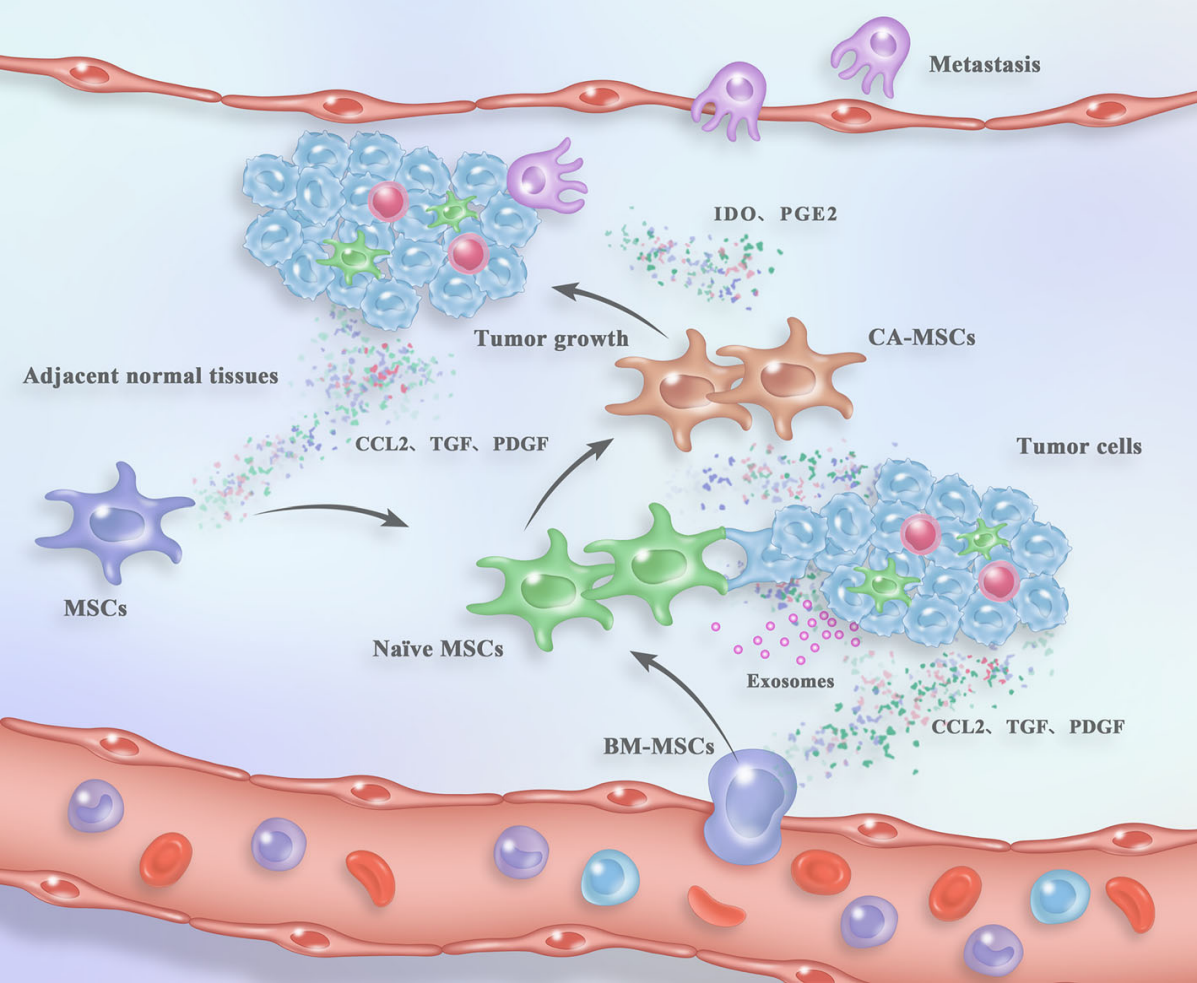
Process of CA-MSC generation and their contribution to tumor fate within the TME. Naïve MSCs are recruited from either the circulation or the adjacent normal tissues in response to the “signals” from tumor tissues. After arriving in the TME, naïve MSCs are “educated” by tumor cells and the TME components in a direct cell-to-cell contact, paracrine or exosome-mediated mode. The “reprogrammed” CA-MSCs facilitate tumor growth and metastasis in turn. For detailed description see the main text.

Collectively, naïve MSCs can be recruited from both the neighboring normal tissues and the distant circulation into tumor mass by tumor cells or other components in the TME. Besides, the nearby non-cancerous stroma has also been considered as the primarily source of CA-MSCs, which may dramatically affect the behavior and fate of tumor (**Figure 1**). Nevertheless, more efforts still need to be devoted to better understand the detailed mechanisms underlying the homing or migration of MSCs toward tumor sites.

## Conversion of Naïve MSCS Into CA-MSCS by Tumor Cells

After the initial stage of tumor development, the tropic naïve MSCs incorporate into tumor mass and their biological properties changed under the influences of tumor cells or non-tumor cells in the TME. Although sharing the similar cell surface markers and plasticity, CA-MSCs display different biology features from naïve MSCs throughout tumor progression. According to emerging evidences, the phenotypes and functions of naïve MSCs can be effectively modulated by tumor cells (8, 52), which give rise to the malignant transition of naïve MSCs into CA-MSCs with striking pro-tumoral property.

A study in prostate cancer reported that tumor cells are important in inducing the transdifferenciation of naïve MSCs into tumor-educated MSCs, which are different from naïve MSCs and can perform potent roles in vascular mimicry and monocyte recruitment (48). Another investigation also showed that naïve MSCs can be “educated” to have pro-metastatic behavior in response to the multiple signals from tumor cells (53). Additionally, a study identified the interactions between primary patient-derived cancer cells and stromal MSCs, and highlighted the specificity of MSC “education/reprogramming” by cancer cells in a tumor-specific manner to support tumor growth (51). In spite of this, understanding the cellular and molecular mechanisms underlying the malignant conversion of tumor tropic MSCs by tumor cells may supply innovative targets for anti-cancer therapy or prognosis in tumor patients.

### Tumor Cells Prompt MSC Malignant Transition by Direct Contact or Paracrine Mode

Hitherto, several literatures have attempted to investigate the mechanisms underlying the malignant transition of naïve MSCs by tumor cells. It was shown in breast cancer that naïve MSCs can be transformed into tumor-forming cells after exposed to tumor cell-derived conditioned medium, aside from direct cell-to-cell contact (52). DNA hypermethylation was revealed in naïve MSCs treated by breast cancer-derived exosomes, which may subsequently contribute to tumor growth and dissemination (52). Another experiment in lung cancer also indentified that conditioned medium from tumor cells participate in promoting naïve MSCs to reprogram toward CA-MSCs, which subsequently promote lung cancer dissemination rather than local growth (33). It was also shown that tumor cells alone can modulate the gene expression profile of naïve MSCs, including IL-6, BST2, ADAMTS12, MX2, LOXL2 and GREM1. In particular, IL6 and BST2 were significantly induced in all naïve MSCs after 3 days’ coculture with primary tumor cells, whereas the expressions of ADAMTS12, MX2, LOXL2 and GREM1 varied and displayed transient induction during the course of the coculture assay (33). However, the cellular and molecular mechanisms for direct cell-to-cell contact between tumor cells and naïve MSCs, or the specificity factors within tumor cell-derived conditioned medium contributing to the introduction of MSCs’ malignant transition still need to be clarified in the future.

### Exosomes Mediate the Malignant Transition of Naïve MSCs by Tumor Cells

Accumulating evidence indicates that exosomes possess an important role in tumor initiation, growth, metastasis and drug resistance (54, 55). By shuttling from one cell to another, exosomes emerged as a novel mode of intercellular communications between tumor cells and tumor-resident MSCs (56–58). Tumor cells may deliver their important “signals” including proteins and nucleic acids (DNA, mRNA and non-coding RNAs) by transporting excessive amount of exosomes, leading to the molecular and genetic changes as well as malignant conversion in naïve MSCs in solid tumors, such as gastric cancer (53, 59) and cholangiocarcinoma (60).

Dramatic changes in the phenotypes and functions of recipient MSCs can be induced by exosome-mediated transfer of “information” from tumor cells, and the “re-programmed” CA-MSCs facilitate tumor growth and metastasis in turn. A study in lymph node metastatic (LNM) gastric cancer focused on the roles of LNM-derived gastric cancer cells (LNM-GCs) in the education of BM-MSCs and identified exosomal Wnt5a as the key protein mediating BM-MSC reprogression by LNM-GCs (53). Moreover, exosome-carried Wnt5a was clarified to elicit activation of YAP signaling, which thereby participates in malignant conversion of BM-MSCs into a tumor-promoting phenotype and incorporating into metastatic microenvironment (53). Another study in cholangiocarcinoma showed that tumor cell-derived extracellular vesicles can “educate” MSCs to induce local microenvironmental changes that facilitate tumour cell growth *via* IL-6/STAT-3 signaling pathway (60). In addition, it was demonstrated that melanoma cell-derived exosomes could induce the formation of a melanoma-like, PD-1 over-expressing population (mMSC^PD-1+^) from naïve MSCs by conveying oncogenic molecular reprogramming (61).

In short, the bi-interactions between MSCs and tumor cells have been proposed to play a critical role in the growth, survival, metastasis and drug resistance of solid tumors (62, 63). But tumor cells alone do not cause the naïve MSCs to acquire all of the features displayed by CA-MSCs (33), indicating the participation of other mediators or factors from the TME in “shaping” the phenotypes and functions of CA-MSCs.

## Conversion of Naïve MSCS Into CA-MSCS by the TME

Possessing important roles in tumor progression, the proportion of CA-MSCs has been demonstrated to dynamically increase throughout the distinct stages of tumor (64). Although tumor cells alone can partly “educate” the naïve MSCs to change their phenotypes and functions, the surrounding microenvironment have also emerged to play a dominant role in the malignant transition of naïve MSCs into tumoral MSCs (65, 66). In various solid tumors, the TME together with tumor cells have been demonstrated to participate in promoting MSC transition into CA-MSCs (67). Tumor cells only cause the recruited naïve MSCs to acquire the biological features of CA-MSCs partially (18, 33). Interactions with microenvironment cells or their factors give rise to both genetic and functional modulations in naïve MSCs (52, 68, 69), which can be effectively transformed into tumor-promoting CA-MSCs.

A study in lung cancer firstly confirmed the role of the TME components in inducing normal tissue-derived MSCs (N-MSCs) to acquire a tumor-associated MSC (T-MSC) expression profile (33). In particular, TGF-β1 and IL-6 generated by the TME components were shown to most likely contribute to the full feature establishment of T-MSCs, which in turn play an important role in tumor metastasis (**Table 2**). Another report in lymphomas demonstrated that L-MSCs can strikingly enhance tumor growth by recruiting monocytes/macrophages (25), suggesting a critical role of monocytes/macrophages in converting the phenotypes and functions of naïve MSCs into CA-MSCs. In addition, TNF-α-pretreated BM-MSCs were shown to mimic L-MSCs in the chemokine profile and ability to promote tumorigenesis (25), suggesting the involvement of TNF-α from monocytes/macrophages in the malignant transition of MSCs (**Table 2**). Likewise, a study in gastric cancer also confirmed the necessity of macrophages in the pro-tumor role of GC-MSCs in a mouse xenograft model with macrophage depletion (15). IL-6 and IL-8 were shown to participate in the interactions between GC-MSCs and macrophages (**Table 2**). Another investigation in gastric cancer focused on tumor-educated neutrophils (TENs) and reported TEN-induced malignant transformation of MSCs by secreting the inflammatory factors including IL-17, IL-23 and TNF-α (70) (**Table 2**).

**Table 2:** The TME components inducing malignant transition of MSCs.

Tumor Types	TME Components	Key Factors	Tumor Fate	Reference
Lung cancer	Non-MSC stromal cells	TGF-β1, IL-6	Metastasis	(33)
Lymphomas	Monocytes/macrophages	TNF-α	Growth	(25)
Gastric cancer	Macrophages	IL-6, IL-8	metastasis	(15)
Gastric cancer	Neutrophils	IL-17, IL-23, TNF-α	Growth, metastasis	(65)

In addition, other key factors in the TME such as hypoxia have also been demonstrated to drive the malignant transformation of naïve MSCs into tumor-promoting MSCs in various types of solid tumors (18, 66, 71). Therefore, the TME components together with their derived factors may have a crucial contribution to the “education” of naïve MSCs.

Notably, the communications between MSCs and non-tumor cells in the TME are complex and the mechanisms deserve to be further investigated in-depth for better understanding tumor progression. A study in gastric cancer demonstrated that miR-155-5p down-regulation induces a phenotypical and functional transition of BM-MSCs into GC-MSC-like cells depending on NF-κB p65 activation (72), suggesting a novel mechanism underlying the CA-MSC “remodeling”. Another report further demonstrated the partial involvement of miR-155-5p in the BM-MSCs “education” by gastric cancer cells (73). However, whether the NF-κB p65/miR-155-5p axis participates in the process of MSC transition, which is modulated by non-tumor cells or their derived factors, still needs to be further investigated. On the other hand, TGF-β secreted by tumor-educated non-tumor cells has been reported by various studies to be responsible for MSC transdifferenciation (45, 74). In spite of this, there is still insufficient information regarding the role of TGF-β signalling pathway in the mechanisms underlying MSC “education” by the non-tumor cells.

Collectively, although non-tumor cells in the TME have been confirmed to play a dominant role in the malignant transition of naïve MSCs, the related cellular and molecular mechanisms are specific and remain ambiguous, which is urgently needed to be illuminated for improving the existing anti-cancer treatments and prognosis.

## Specificities of the Generating Process of CA-MSCS

In response to the “cues” from tumor, the recruited MSCs can acquire biological characteristics of CA-MSCs *via* a direct cell-to-cell contact, paracrine or exosome-mediated mode. Although the generating process of tumor-promoting CA-MSCs has not yet been clearly elucidated, multiple reports have demonstrated the specificities of MSC transition, by which naïve MSCs can be “re-programmed” (i) in a tumor type-dependent manner; (ii) with a relatively continued stable phenotype and (iii) with different kinetics throughout tumor progression. The specificities of CA-MSC generation further advance our understanding of kinetic changes on the genetic and phenotypic properties of tumor resident MSCs.

### Naïve MSCs Are Reprogrammed Into CA-MSCs in a Tumor-Specific Manner

The generating progress of CA-MSCs may vary depending on the tumor type they reside in. A study isolated CA-MSCs from gastric cancer and lung cancer simultaneously, and identified the differences between GC-MSCs and LC-MSCs (51). In comparison with LC-MSCs, GC-MSCs possess high expressions of HGF, CD146 and ABCG1 transporter (51), as well as higher multi- potency, suggesting specificity of CA-MSC generation to support tumor growth. It was also shown that the ovarian TME cannot reprogram BM-MSCs into CA-MSCs although breast cancer TME display an effectively role in MSC transformation, suggesting that the tumor-mediated MSC conversion is tumor-type dependent (18). Another investigation also observed the tumor type-specificity in MSC re-programming. The influence of tumor cells in MSC transition vary according to the tumor cell properties and MSCs acquire different characteristics according to the tumor type and the corresponding TME (33).

### CA-MSCs Obtain a Relatively Continued Stable Phenotype

After malignant transition, the re-programmed CA-MSCs appear to be relatively stable in their phenotypes and biological characteristics. The maintaining of CA-MSCs’ properties in co-culture systems is not dependent on the continued presence of tumor cells (18, 33). Thus, this relatively continued stable phenotype of tumor-”educated” CA-MSCs makes them a more amenable target for anti-cancer therapy other than genetically labile tumor cells.

### The Generating Process of CA-MSCs Is Dynamic

The modulation of MSCs’ phenotype by either tumor cells or the TME appears to be dynamic at distinct stages of tumor progression. As a multiple population, the subsets of CA-MSCs are dynamic and CA-MSCs should be taken into consideration their distinct states with variable gene expression and functions throughout the tumor pathophysiology (64, 75).

Nevertheless, further studies are still needed to search for more specificities of MSC transition in tumor development and progression, which may enhance our understanding of the generating process of CA-MSCs in solid tumors.

## Conclusions

As one of the key stromal cells within tumor niche, CA-MSCs have been isolated from various types of solid tumors and identified to play important roles in tumorigenesis, metastasis and drug resistance (27, 76, 77). Compared with naïve MSCs, CA-MSCs display several unique phenotypes and more potent pro-tumorigenic functions, which are considered as a key regulator of tumor fate (77–79). Hitherto, the origins and generating progress of CA-MSCs still remain ambiguous.

In response to the “cues” from tumor cells or the TME, naïve MSCs are recruited from both the adjacent normal tissues (16) and the distant circulation (25) into tumor site. Moreover, the nearby non-cancerous stroma is positioned as the primarily source of naïve MSCs. Although the origins and tumor-tropic progress of MSCs have been clarified to some extent for our understanding, detailed mechanisms underlying the tropism and migration of MSCs towards tumor should be further investigated. After arrived into the TME, the phenotypes and functions of naïve MSCs are “shaped” by the tumor cells and other components in the microenvironment in a direct cell-to-cell contact, paracrine or exosome-mediated mode (**Figure 1**) (52, 80). In addition, the conversion of naïve MSCs into CA-MSCs has been demonstrated to be a specific progression, which is tumor-type dependent, malignantly stable, and dynamic at distinct stages of tumorigenesis and progression (18, 33). However, more in-depth studies are still needed for discovering the generating process or transition of CA-MSCs in solid tumors, which may provide biomarkers or potential targets such as CA-MSC upstream or downstream modulators, for cancer prognosis and treatments.

## Perspectives

As one of the key components in tumor stroma, CA-MSCs display a potent role in the development and progression of solid tumors, such as gastric cancer, ovarian cancer and lung cancer. Understandings of the origins and generation of CA-MSCs will facilitate the development of novel targets for anti-cancer treatments and prognosis. After tropism into tumor site from the circulation or adjacent normal tissues, the infiltrated naïve MSCs can be “educated” into CA-MSCs by tumor cells and other components in the TME. However, more efforts still need to be devoted to better elucidate the cellular and molecular mechanisms underlying MSC migration and malignant transition, which may provide more promising targets for tumor therapy and prognosis

## Author Contributions

WL, JY, PZ and HL collected the related paper and drafted the manuscript. WL and SZ participated in the design of the review. All authors contributed to the article and approved the submitted version.

## Funding

This review was supported by the National Natural Science Foundation of China (Grant No. 81402280), the Foundation of Open Project from Jiangsu Key Laboratory (Grant No. XZSYSKF20200001), the Jiangsu Postdoctoral Research Foundation (Grant No. 1501079A) and the Doctoral Foundation from the First People’s Hospital of Lianyungang (Grant No. BS1503).

## Conflict of Interest

The authors declare that the research was conducted in the absence of any commercial or financial relationships that could be construed as a potential conflict of interest.

## Publisher’s Note

All claims expressed in this article are solely those of the authors and do not necessarily represent those of their affiliated organizations, or those of the publisher, the editors and the reviewers. Any product that may be evaluated in this article, or claim that may be made by its manufacturer, is not guaranteed or endorsed by the publisher.
